# Parallel Arousal Pathways in the Lateral Hypothalamus

**DOI:** 10.1523/ENEURO.0228-18.2018

**Published:** 2018-08-21

**Authors:** Jaime E. Heiss, Akihiro Yamanaka, Thomas S. Kilduff

**Affiliations:** 1 Biosciences Division, Center for Neuroscience, SRI International, Menlo Park, CA 94025; 2 Department of Neuroscience II, Research Institute of Environmental Medicine, Nagoya University, Furo-cho, Chikusa-ku, Nagoya, 464-8601, Japan

**Keywords:** arousal, hypocretin, orexin, sleep, wakefulness

## Abstract

Until recently, hypocretin (Hcrt) neurons were the only known wake-promoting neuronal population in the lateral hypothalamus (LH), but subpopulations of inhibitory neurons in this area and glutamatergic neurons in the nearby supramammillary nucleus (SuM) have recently been found that also promote wakefulness. We performed chemogenetic excitation of LH neurons in mice and observed increased wakefulness that lasted more than 4 h without unusual behavior or EEG anomalies. The increased wakefulness was similar in the presence or absence of the dual orexin receptor blocker almorexant (ALM). Analysis of hM3Dq transfection and c-FOS expression in LH inhibitory neurons and in the SuM failed to confirm that the increased wakefulness was due to these wake-promoting populations, although this possibility cannot be completely excluded. To evaluate the relationship to the Hcrt system, we repeated the study in *Orexin-tTA* mice in the presence or absence of dietary doxycycline (DOX), which enabled us to manipulate the percentage of Hcrt neurons that expressed hM3Dq. In DOX-fed mice, 18% of Hcrt neurons as well as many other LH neurons expressed hM3Dq; these mice showed a profound increase in wake after hM3Dq activation even in the presence of ALM. In mice switched to normal chow, 62% of Hcrt neurons expressed hM3Dq along with other LH cells; chemogenetic activation produced even more sustained arousal which could be reduced to previous levels by ALM treatment. Together, these results indicate an LH neuron population that promotes wakefulness through an Hcrt-independent pathway that can act synergistically with the Hcrt system to prolong arousal.

## Visual Abstract

**Figure:**
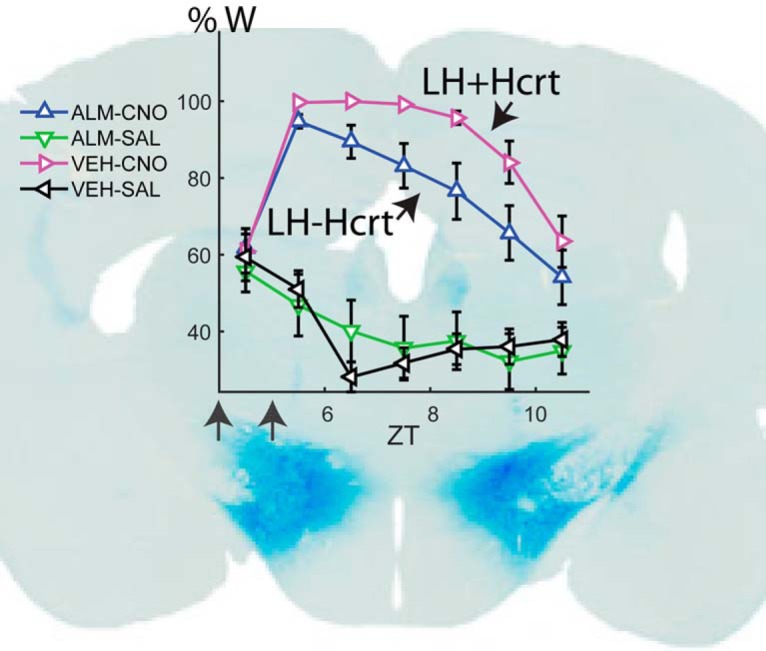

## Significance Statement

Despite nearly a century of evidence suggesting that the lateral hypothalamus (LH) contains neurons essential to sustain wakefulness, the primary wake-promoting cells in this brain region studied to date have been the hypocretin (Hcrt)/orexin neurons. Here, we show that chemogenetic excitation of LH neurons can induce sustained wakefulness even in absence of Hcrt signaling and that concurrent Hcrt neuron activation can prolong arousal. These results indicate the existence of parallel arousal pathways in the LH and suggest a new therapeutic target for disorders of excessive sleepiness such as hypersomnia and narcolepsy.

## Introduction

The lateral hypothalamus (LH) has been thought to play a critical role in sleep-wake regulation since the early studies of Von Economo (1930). Lesions and acute inhibition of LH neurons are known to increase sleep time (Ranson, 1939; Nauta, 1946; de Ryck and Teitelbaum, 1978; Shoham and Teitelbaum, 1982; Cerri et al., 2014) while excitation produces arousal and increases in activity (Królicki et al., 1985; Sinnamon et al., 1999; Alam and Mallick, 2008; Li et al., 2011). Several studies have identified wake-active neurons in the LH (Alam et al., 2002; Koyama et al., 2003; Lee et al., 2005), but their neurochemical identity is unknown. More recently, inhibitory LH neuronal populations have been identified that induce wakefulness on optogenetic or chemogenetic stimulation (Herrera et al., 2016; Venner et al., 2016). The wake-promoting LH cell population that has been studied most extensively to date are the hypocretin/orexin (Hcrt) neurons (de Lecea et al., 1998; Peyron et al., 1998; Kilduff and Peyron, 2000; Gerashchenko et al., 2001; Bonnavion and de Lecea, 2010). These cells project to several wake-promoting areas of the brain including the basal forebrain (BF), the tuberomamillary nucleus (TMN), and the locus coeruleus (LC), where they can release the Hcrt peptides, glutamate, dynorphin, and perhaps other neurotransmitters (Torrealba et al., 2003; Eriksson et al., 2004; Schöne et al., 2012, 2014). In this regard, the Hcrt neurons have been proposed to act like a stabilizer of a flip-flop switch that controls behavioral state (Saper et al., 2005).

Narcolepsy is a neurologic disease characterized by the extensive loss of Hcrt neurons without degeneration of neighboring cells (Kilduff and Peyron, 2000; Thannickal et al., 2000). Narcoleptic patients are unable to sustain long periods of consolidated wakefulness and must nap frequently; some patients also experience attacks of cataplexy which greatly affects their quality of life (Kornum et al., 2017). However, total sleep time in untreated narcoleptic patients as well as in mouse models of narcolepsy does not differ from controls over the 24-h period (Dantz et al., 1994; Hara et al., 2001; Tabuchi et al., 2014), a phenotype that is distinct from the lethargy observed after LH lesions or acute LH neuronal inhibition. Chemogenetic excitation of Hcrt neurons in mice (Sasaki et al., 2011) produced a 30% increase in wake time during the first hour of stimulation compared to controls but no significant difference was observed after the second hour, which also differs from the effects of LH excitation cited above. Thus, Hcrt neuron stimulation and ablation studies do not fully recapitulate the effects on wake and sleep observed after manipulations of LH neuron activity, indicating that there are likely other wake-promoting neuronal populations in the LH that may be involved in sleep/wake regulation.

To determine whether LH stimulation has wake-promoting effects distinct from those exhibited by Hcrt neuron stimulation, we performed chemogenetic excitation of LH neurons while conducting EEG and EMG recordings of the transfected mice. We then evaluated the effect of time-of-day on LH-mediated wake promotion and whether Hcrt signaling was necessary to evoke arousal during LH activation. Finally, we combined transfection of LH neurons with a diet-dependent expression of hM3Dq in Hcrt neurons to test whether the Hcrt and non-Hcrt arousal pathways are parallel or overlapping. Our results show that LH neuronal stimulation evokes profound but physiologic arousal independent of Hcrt signaling and that concurrent activation of Hcrt and non-Hcrt LH neurons has synergistic effects on arousal that are attenuated in presence of the dual orexin receptor antagonist almorexant (ALM). These results indicate the existence of an LH arousal pathway that can be activated even when Hcrt signaling is pharmacologically blocked, suggesting a novel target for treatment of sleep/wake disorders such as narcolepsy.

## Materials and Methods

### Animals

Adult *Gad2-IRES-Cre;R26R-EYFP* (Jax stock #010802 and #006148; *n =* 6 males and 4 females, two to four months old) and *Orexin-tTA* mice (C57BL/6-Tg(orexin/tTA)G5/Yamanaka; *n =* 21 males, two to four months old), which express the tetracycline transactivator (tTA) exclusively in the Hcrt neurons (Tabuchi et al., 2014), were singly-housed under a 12/12 h light/dark cycle with *ad libitum* access to food and water in temperature (24 ± 2°C) and humidity (50 ± 20% relative humidity) controlled rooms. Sixteen of the *Orexin-tTA* mice were reared with chow that included doxycycline (DOX). An additional cohort of C57BL/6J WT mice (Jax stock #000664; *n = 4* males and 4 females, three to six months old) were housed under the same conditions and used to assess the effects of clozapine-N-oxide (CNO) treatment on sleep/wake in naïve mice (Table 1). All studies were conducted in accordance with the Guide for the Care and Use of Laboratory Animals and were approved by the Institutional Animal Care and Use Committee at SRI International. Every effort was made to minimize animal discomfort throughout these studies.

**Table 1.** Summary of experimental treatments

Experiment	Strain	Sex	*N*	Number of treatments	Circadian phase	Results
CNO effect	*Gad-Cre;R26R-EYFP*	M and F	9	2	Active	Figs. 2, 3
ALM effect	*Gad-Cre;R26R-EYFP*	M and F	9	2	Active	Not shown
CNO control	C57BL/6J	M and F	8	2	Inactive	Fig. 3*B*
ALM and CNO interaction	*Gad-Cre;R26R-EYFP*	M and F	10	4	Inactive	Figs. 4, 5
ALM and CNO interaction	*Orexin-tTA* (DOX+)	M	11	4	Inactive	Fig. 8*C*,*E*
ALM and CNO interaction	*Orexin-tTA* (DOX-)	M	13	4	Inactive	Fig. 8*D*,*F*

All Gad-Cre;R26R-EYFP mice were Cre^+^/R26^+^ except one Cre^+^/R26^-^ male mouse.

### Surgical procedures

Under isoflurane anesthesia, *Gad2-IRES-Cre;R26R-EYFP* and *Orexin-tTA* mice were injected bilaterally into the LH (-1.4 AP, 1 ML relative to bregma, and -5 DV from pia) with 340 nl of an AAV encoding hM3Dq-mCherry [AAV-TetO-hM3Dq-mCherry (Sr10); titer 2 × 10^11^; Nagoya University, Japan] using a Picospritzer II (Parker Instrumentation) connected to a BK Precision 4003A Pulse Generator (B&K Precision Corporation) which delivered 5- to 20-ms pulses of pressurized air every 2 s into a glass micropipette for 5–10 min. After a recovery period of at least two weeks, injected mice as well as naïve C57BL/6J mice underwent surgery for implantation of EEG and EMG leads by cementing a reference EEG screw over the occipital bone, one in the left frontal bone (1 AP, 1 ml), and another over the parietal bone (-2 AP, 2 ml). EMG electrodes were two stainless steel-coiled wires anchored to the neck musculature. A five-pin connector was connected to the EEG and EMG leads and cemented to the skull for conventional tethered EEG/EMG recordings.

### Experimental procedures

To allow acclimation to the recording apparatus, mice were connected to a tether that was attached to a commutator (Pinnacle Technology Inc.) for at least 5 d before experiments. The commutator allowed the mice to move freely in their home cages. For acclimation to the dosing procedure, mice underwent three intraperitoneal injections of saline (SAL) on different days before initiation of the dosing study. Experiments occurred no sooner than 21 d after the EEG/EMG implantation surgery. EEG and EMG data were collected at 800 Hz and digitally band-passed at 0.5–300 Hz for *Gad2-IRES-Cre;R26R-EYFP* and WT mice, and at 500 Hz and digitally band-passed at 0.5–200 Hz for *Orexin-tTA* mice using a TDT RZ2 system connected to a 96-channel PZ2 amplifier and a low impedance RA16LI-D headstage (Tucker-Davis Technologies). To monitor activity, infrared analog video was collected using CNB B1760N4.3 cameras (CNB Technology Inc.) and digitized at 30 fps using a Q-See QT5616 system (Q-See). All animals underwent an undisturbed 24-h baseline recording in their home cages before dosing experiments.

### Dosing procedures

CNO (catalog #4936, Tocris Bioscience), the ligand for the modified muscarinic receptor hM3Dq, was injected intraperitoneally at 3 mg/kg in SAL solution at a concentration of 0.5 mg/ml in all experiments. To block Hcrt signaling, the dual orexin receptor antagonist ALM, synthesized at SRI International (Black et al., 2013), was injected intraperitoneally at a concentration of 200 mg/kg in vehicle (VEH) consisting of 1.25% hydroxypropyl methyl cellulose (SKU 09963), 0.1% dioctyl sodium sulfosuccinate (SKU 323586), and 0.25% methyl cellulose (SKU 274429) in water (all from Sigma-Aldrich) after vortexing a solution of 30 mg/ml for 2 h. Dosing sessions were at least 4 d apart and at least 7 d elapsed between CNO dosings.

In *Gad2-IRES-Cre;R26R-EYFP* mice, dosing occurred either at (Zeitgeber Time (ZT) where ZT12 is lights off and ZT0 is lights on) and EEG/EMG was recorded for the subsequent 12 h or at ZT4 and the EEG/EMG was recorded for 9 h starting at ZT3. In *Orexin-tTA* mice, dosing studies were also performed at ZT4 and EEG/EMG was recorded from ZT4 to ZT11. For mice belonging to both the DOX(+) and DOX(-) cohort (*n =* 10), the DOX-containing chow was replaced by normal chow at least three weeks before the dosing study was repeated under the DOX(-) condition.

### Locomotor activity data collection and analysis

Synchronization of EEG/EMG data and time-stamped video was achieved by synchronizing the Q-See system’s clock to the TDT computer’s clock and manually entering the start and end time of all behavioral videos. The position of the mice was detected automatically using Noldus EthoVision XT 11.5 software (Noldus Information Technology). To calculate the speed of the mice in Figure 3*F*,*G*,[AQ4] after manually curating the automatic tracking of the mouse’s position, we obtained a matrix with the *X* and *Y* coordinates of each mouse 30 times/s as well as a vector with the relative time of each data point. A median filter and then an average filter with a moving window of 10 time points and step of one time point was applied to both sets of coordinates and the Euclidean distance with respect to the origin of the coordinate system was obtained for every time point. The distance traveled was calculated as the absolute value of the difference between the calculated distance and the distance from the first data point. Finally, the speed was calculated as the absolute difference between two consecutive points of the distance traveled, divided by the difference in time between the two consecutive points. All data were processed using custom scripts created in MATLAB 2015b (MathWorks).

### EEG/EMG data collection and analysis

Using the criteria described previously (Morairty et al., 2013; Dittrich et al., 2015), EEG/EMG data were visually scored as wake (W), non-rapid eye movement sleep (NREM), or rapid eye movement sleep (REM) in 4-s epochs. A bout of W, NR, or REM was defined as three consecutive 4-s epochs (i.e., 12 s) within the same state. For quantitative EEG analysis, the power spectrum was calculated for each 4-s artifact-free epoch using the squared amplitude coefficients of the fast Fourier transform and the mean power in the δ, 0.5–4 Hz; low θ (Lθ), 4–8 Hz; high θ (Hθ), 8–10 Hz; low γ (Lγ), 15–50 Hz; high γ (Hγ), 60–80 Hz; and very high γ (Vhγ), 90–200 Hz range. To avoid 60-Hz artifact, the spectral power values at 60 ± 1 Hz and the harmonics at 120 and 180 Hz were replaced by a linear interpolation from the values at 59 and 61, 119 and 121, and 179 and 181 Hz. Normalized W power was obtained by dividing the mean power spectrum during W by the mean power in W during the same time period of a baseline recording.

### Experimental design and statistical analyses

Table 1 summarizes the six dosing experiments that were conducted. Results obtained from male mice were similar to those of female mice and thus data from both sexes were grouped. For statistical analysis, we performed two-way repeated measures ANOVA using Sigmaplot (Systat Software Inc.) followed by Bonferroni-corrected *post hoc t* tests to identify effects of the different treatments on sleep architecture across time after dosing. To assess sleep rebound effects after the prolonged wakefulness induced by LH neuron activation, we considered all time points following the first hour after dosing when the hourly average NREM sleep time was at least 20%. For comparisons involving only one factor, we used the MATLAB function anova1 followed by Tukey’s *post hoc* test with Bonferroni correction to identify individual differences between two treatments. To compare the effect of two factors on two different populations, we performed an unbalanced two-way ANOVA using the MATLAB function anovan. For estimation of whether two datasets had the same probability distribution, we used the two-sample Kolmogorov–Smirnov goodness-of-fit hypothesis test with the MATLAB function kstest2. The Bonferroni correction was also used here for pairwise comparison of more than two distributions. For calculation of Pearson’s correlation coefficient (*r*), we used the MATLAB function corrcoef and a significance threshold of *p* < 0.05. To determine the difference between two populations of similar size and estimation of the standard error over the mean associated with a 95% confidence level, we used the formula

$$m_{1}-m_{2}\pm \frac{\left(z*\sqrt{\frac{s_{1}^{2}}{n_{1}}+\frac{s_{2}^{2}}{n_{2}}}\right)}{\sqrt{min(n_{1},}n_{2})}$$

where *z* = 1.96 and *m*, *n* and *s* are the mean, size, and SD of the respective population.

### Histology

At the end of the experiments, mice were anesthetized with an intraperitoneal overdose of euthanasia solution (Beuthanasia-D, Intervet) and intracardially perfused with PBS (0.01 M, pH 7.2–7.4) followed by 4% paraformaldehyde (PFA) in PBS. Brains were postfixed in PFA overnight at 4°C and then cryoprotected in 30% sucrose in PBS at 4°C. Coronal 30-µm sections were cut on a freezing microtome. Slices separated by either 180 or 90 µm (i.e., either one in six or one in three series) were processed for immunohistochemistry after three washes in PBS for 10 min each and then transferred to a meshed well where they remained during the entire immunohistochemistry process to prevent tissue damage. For diaminobenzidine (DAB) staining of c-FOS and mCherry-transfected neurons, slices were quenched in 0.3% H_2_O_2_ in PBS for 30 min before incubation with primary antisera. Slices were incubated in blocking solution (1% Triton X-100 and 5% donkey serum in PBS) at room temperature (RT) for 1 h and then incubated overnight at 4°C in blocking solution containing the primary antibody. After five washes in PBS for 5 min each, slices were incubated for 1 h at RT in the secondary antibody and washed five times in PBS. For DAB staining, the avidin-biotin-peroxidase system Elite PK-6100 combined with DAB SK-4100 (Vector Laboratories) was used according to the directions. Black c-FOS labeling was obtained by adding nickel chloride to the DAB mix (1.6%).

The primary antisera used (all at 1:2000 dilution) were goat anti-orexin-A (Santa Cruz Biotechnology catalog #sc-8070; RRID:AB_653610) and goat anti-orexin-B (Santa Cruz Biotechnology catalog #sc-8071; RRID:AB_653612), c-FOS rabbit antibody (226003, Synaptic Systems GmbH; RRID: AB_2231974), chicken anti-GFP (which also recognizes EYFP, Abcam catalog #ab13970; RRID: AB_300798), rabbit anti-adenosine deaminase (ADA; catalog #AB176, EMD Millipore; RRID: AB_2222916), RFP rabbit antibody (600-401-379, Rockland; RRID: AB_2209751), and rat anti-mCherry antibody (1:1000, catalog #M11217, Thermo Fisher Scientific, RRID: AB_2536611).

Secondary fluorescent antisera used were donkey anti-goat (A-11056; RRID: AB_142628) Alexa Fluor 546, donkey anti-rat (catalog #ab150155, Abcam, RRID: N.A.) Alexa Fluor 647, donkey anti-rabbit (A-10042; RRID: AB_2534017), Alexa Fluor 568, donkey anti-rabbit (catalog #A-21206; RRID: AB_2535792), Alexa Fluor 488 and goat anti-chicken (A-11039; RRID: AB_142924), and Alexa Fluor 488 (Thermo Fisher Scientific). Secondary biotinylated antisera used was donkey anti-rabbit (711-065-152; RRID: AB_2340593, Jackson ImmunoResearch Laboratories, Inc.). All secondary antisera were used at a 1:500 dilution. All cell counts were done under double blind conditions.

## Results

### Transfection of LH cells

**Figure 1.**
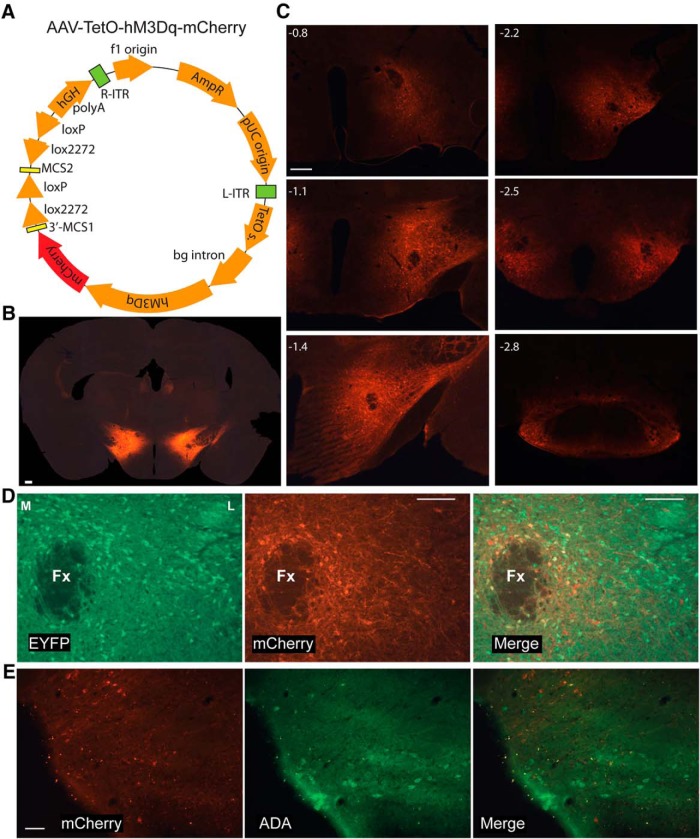
Expression of hM3Dq in the LH. ***A***, Schematic of the AAV used. ***B***, AAV-injected *Orexin-tTA* mice exhibited widespread neuronal expression of hM3Dq-mCherry despite the presence of DOX in the chow. ***C***, Despite the absence of tTA, *Gad2-IRES-Cre;R26R-EYFP* mice injected with AAV-TetO-hM3Dq-mCherry exhibited a pattern of hM3Dq-mCherry expression that was similar to DOX-fed *Orexin-tTA* mice. Scale bar in ***B***, ***C*** = 250 µm. Numbers in the upper left in ***C*** denote approximate AP distance from bregma (mm). ***D***, Photomicrographs showing GAD2-EYFP^+^ neurons lateral to the fornix (Fx, left), hM3Dq-mCherry transfected neurons (center) and double-labeled neurons in yellow after merging both images (right). Scale bar = 100 µm. M and L denote medial and lateral. ***E***, Photomicrographs showing ADA^+^ neurons in TMN (left), hM3Dq-mCherry transfected neurons (center), and merged image (right) showing the absence of double-labeled neurons. Scale bar = 50 µm.

With the intention of transfecting only Hcrt neurons, AAV10-TetO-hM3Dq-mCherry (340 nl) was injected bilaterally into the LH of *Orexin-tTA* mice fed with DOX (Fig. 1*A*,*B*). Considerable ectopic expression was observed although expression of the transgene encoded in this AAV should be dependent on the presence of the tTA and expressed only in the absence of DOX. Because TetO includes a minimal promoter sequence, TetO-linked transgenes can be expressed without tTA if a high enough AAV copy number transfects the cells of interest. To determine whether inhibitory as well as excitatory cells were transfected under these conditions, the same volume of AAV was injected at the same coordinates in *Gad2-IRES-Cre;R26R-EYFP* mice (Taniguchi et al., 2011) in which the glutamate decarboxylase 2 (GAD2) gene is tagged with yellow fluorescent protein (YFP). Coronal 30-µm sections were cut through the hypothalamus and every 6th section was processed for mCherry and EYFP immunohistochemistry; amplification of EYFP using Alexa Fluor 488 as a secondary antibody was used to label inhibitory neurons in green. The ectopic expression of hM3Dq-mCherry found in *Gad2-IRES-Cre;R26R-EYFP* mice was similar to that observed in *Orexin-tTA* mice. Injections primarily diffused in the ventral and caudal directions and consistently produced neuronal expression of hM3Dq-mCherry in both *Gad2-IRES-Cre;R26R-EYFP* and *Orexin-tTA* mice. Figure 1*C* shows that transfected neurons were detected from -0.8 to -2.8 mm relative to bregma. We counted 11,611 transfected neurons from 10 *Gad2-IRES-Cre;R26R-EYFP* mice; 29.9 ± 2.6% of them were GAD2-positive (mean ± SEM), indicating that only a minority of transfected cells were inhibitory. Figure 1*D* shows EYFP-labeled inhibitory neurons around the fornix at ∼1.7 mm from bregma (left), hM3Dq-mCherry expression in the same area (center), and the merged image (right) revealing the rare occurrence of double-labeled (yellow) cells.

To verify that histaminergic (HA) neurons in the tuberomammillary nucleus (TMN) were not transfected by the AAV, we labeled these neurons with rabbit anti-ADA and an anti-rabbit antibody conjugated with Alexa Fluor 488 together with rat-mCherry antibody and anti-rat antibody conjugated with Alexa Fluor 647. We counted unilaterally the number of ADA-labeled cells from a slide corresponding to the TMN at ∼2.5 mm in the A-P direction from bregma in eight AAV-transfected *Gad2-IRES-Cre;R26R-EYFP* mice. Out of a total of 177 ADA-labeled cells, only three also expressed mCherry, averaging 1.8 ± 0.9% of TMN-HA cells per mouse. Therefore, we conclude that TMN-HA cells were not significantly transfected by the AAV. Figure 1*E* illustrates an example of the lack of mCherry on TMN-HA neurons.

### LH activation induces several hours of continuous wakefulness

**Figure 2.**
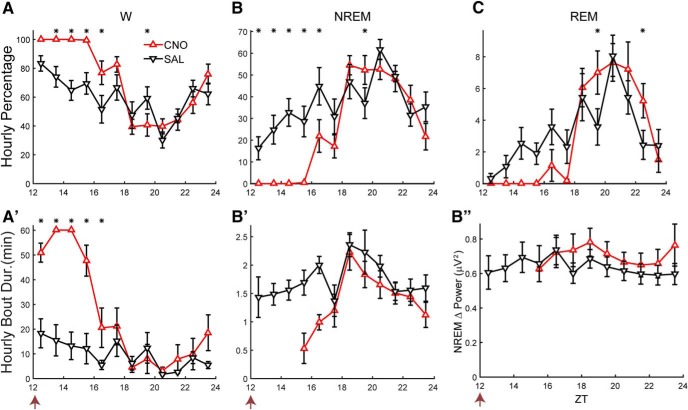
LH activation induces continuous wakefulness for at least 4 h. Hourly percentage of time spent in W (***A***), NREM (***B***), and REM sleep (***C***) and hourly bout duration for W (***A’***) and NREM (***B’***) after injection of either CNO (red) or SAL (black). Asterisks denote significant difference between CNO and SAL treatment for the respective hour when a significant treatment × time interaction was found (*p* ≤ 0.047, *n =* 9; two-way repeated measures ANOVA, Bonferroni *post hoc t* test). ***B’’***, No significant change in NREM δ power was observed after the prolonged W induced by CNO injection (n.s., *n =* 6, two-way repeated measures ANOVA). Arrow at bottom left of abscissa indicates time of dosing at ZT12.

Because the AAV-TetO-hM3Dq-mCherry construct allowed targeting of a broad population of LH cells for experimental manipulation, this AAV was injected in the LH of *Gad2-IRES-Cre;R26R-EYFP* mice (*n =* 10) that were subsequently implanted for EEG and EMG recordings. To determine whether activation of LH neurons had an effect on sleep/wake, mice were dosed at ZT12 with either CNO (0.5 mg/ml, 3 mg/kg) or SAL. Figure 2 shows that *Gad2-IRES-Cre;R26R-EYFP* mice spent close to 100% of the time awake for the first 4 h after CNO administration (Fig. 2*A*) and almost no time in either NREM (Fig. 2*B*) or REM sleep (Fig. 2*C*) during that period. ANOVA revealed significant interaction of treatment × time on the percentages of time spent in W (*F*_(11,88)_ = 5.2, *p* < 0.001), NREM (*F*_(11,88)_ = 5.1, *p* < 0.001), and REM sleep (*F*_(11,88)_ = 2.6, *p* = 0.007) as well as W bout duration (*F*_(11,88)_ = 8.7, *p* < 0.001, *n =* 9, two-way repeated measures ANOVA; Fig. 2*A’*). The hours that were significantly different between treatments are denoted by asterisks (*p* < 0.05, Bonferroni *post hoc t* test); significant wake-promoting effects were observed for at least 5 h after CNO injection compared to SAL. Some short NREM sleep bouts were detected during the fourth hour after injection, but the absence of NREM in all subjects for the first several hours precluded statistical comparisons of sleep bout durations (Fig. 2*B’*).

To determine whether a homeostatic response occurred in response to the prolonged wakefulness induced by LH neuron activation, NREM δ power (NRD; defined as spectral power in the 0.5- to 4-Hz range during NREM sleep) was compared from the first time point after dosing when the hourly average NREM time was at least 20% (which occurred after ZT18–ZT19) until the end of the recording (Fig. 2*B’* *’*). Despite the prolonged W and reduction of NREM and REM sleep evident in Figure 2*A–C*, no difference in NRD between CNO and SAL groups or treatment × time interaction was observed for the six mice that exhibited NREM bouts during all hours when NREM time was at least 20% (*F*_(1,5)_ = 3.5 and *F*_(5,25)_ = 0.9, n.s., *n =* 6, two-way repeated measures ANOVA).

**Figure 3.**
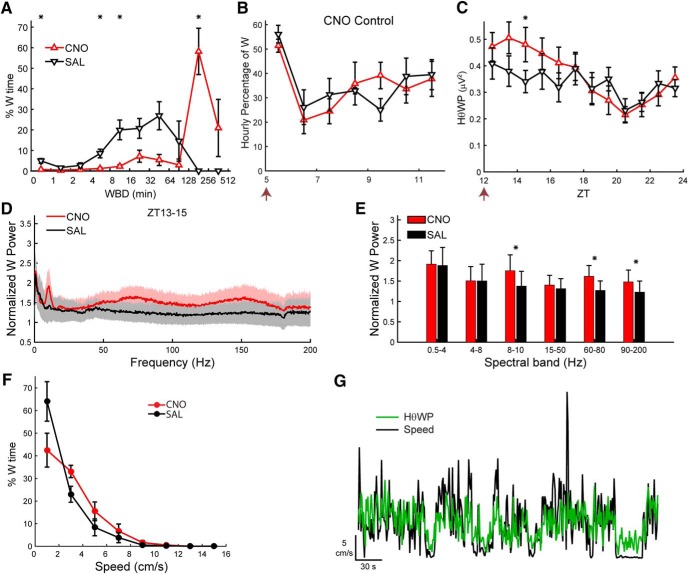
Characterization of CNO-induced wakefulness. ***A***, Time-weighted frequency histograms showing the proportion of wake bouts of each duration (WBD) relative to the total amount of wakefulness from ZT12 to ZT17 after CNO or SAL treatment in *Gad2-IRES-Cre;R26R-EYFP* mice. Asterisks denote significant differences (*p <* 0.04, *n =* 9, *t* test with Bonferroni correction for multiple comparisons). ***B***, Hourly percentage of time spent in W for naïve WT mice injected with CNO or SAL during the inactive phase. ***C***, HθWP in the dark period after CNO or SAL dosing of *Gad2-IRES-Cre;R26R-EYFP* mice at ZT12. Asterisk denotes a significant difference for the respective hour when a significant treatment × time interaction was found (*p* = 0.046, *n =* 9; two-way repeated measures ANOVA, Bonferroni *post hoc t* test). ***D***, Normalized EEG power spectrum during W averaged from ZT13 to ZT15 after dosing of *Gad2-IRES-Cre;R26R-EYFP* mice with CNO (red) or SAL (black) at ZT12. ***E***, Mean power for EEG spectral bands of *Gad2-IRES-Cre;R26R-EYFP* mice after dosing with either CNO or SAL, normalized by baseline. Asterisk denotes a significant difference for the respective band when ANOVA revealed a significant treatment × power band (*p* = 0.002, *n =* 9; two-way repeated measures ANOVA, Bonferroni *post hoc t* test). ***F***, Percentage of time mice spent moving at different speeds from ZT12 to ZT14 during W. ***G***, Example of simultaneous recordings of mean speed per epoch (black trace) and scale-adjusted HθWP (green trace).

Because strong wake-promoting effects after LH activation were observed for 5 h after dosing, the distribution of W bout durations during this period was compared between the CNO and SAL groups (Fig. 3*A*). The percentage of time spent in W bouts of 0- to 1- and 4- to 16-min duration was lower after CNO than after SAL injection; in contrast, nearly 80% of the total W time after CNO injection was spent in W bouts >128 min in duration, which were nonexistent after SAL injection (*p* < 0.05, *n =* 9; Student’s *t* test with Bonferroni correction for multiple comparisons). Nonetheless, comparison of the distribution of W bout durations between CNO and SAL treatments did not quite reach statistical significance (*p* = 0.051, *n =* 83 and 274 bouts for CNO and SAL treatments, respectively; two-sample Kolmogorov–Smirnov goodness-of-fit hypothesis test).

As a control for CNO injection, a cohort of naïve C57BL/6J mice (*n =* 8) were instrumented for EEG/EMG recordings and, after recovery from surgery and acclimation to the experiment, received either CNO (3 mg/kg, i.p.) or SAL during the inactive phase at ZT5 when any increase in W time should be readily evident. Figure 3*B* presents the hourly percentage of W time after dosing for both treatments. No significant difference was found in the percentage of time in any state, bout duration or any frequency band in the power spectrum between the two treatment conditions.

Because *Gad2-IRES-Cre;R26R-EYFP* mice remained continuously awake for several hours after CNO injection, we analyzed the EEG power spectrum and no grossly abnormal patterns in EEG activity were detected (Fig. 3*C–E*). However, a pronounced peak occurred in the 8- to 10-Hz range (Hθ) during W, a higher frequency than the θ peak observed during REM sleep which is centered around 7 Hz. Figure 3*C* shows a trend toward elevated Hθ power during W (HθWP) for 5 h after dosing. Statistical analysis confirmed a significant treatment × time interaction (*F*_(11,88)_ = 4.2, *p* < 0.001, *n =* 9, two-way repeated measures ANOVA), although *post hoc* analysis determined that ZT14–ZT15 was the only hour during which HθWP was significantly elevated (Fig. 3*C*). The normalized mean power spectrum between ZT13 and ZT15 during W was slightly higher after CNO dosage for most frequencies (Fig. 3*D*). To quantify the differences in normalized power between conditions, we grouped the frequencies into the bands described in the Materials and Methods: δ, Lθ, Hθ, Lγ, Hγ, and Vhγ (Fig. 3*E*). A significant interaction of treatment × power band was observed (*F*_(5,40)_ = 4.5, *p* = 0.002, *n =* 9, two-way repeated measures ANOVA). *Post hoc* analysis showed significantly elevated power during ZT13–ZT15 for Hθ, Hγ, and Vhγ, which were 27%, 28%, and 21% greater, respectively, after CNO injection.

### LH activation induces a physiologic increase in locomotor activity

To determine whether LH activation by CNO injection induced abnormal behavior, the activity of nine *Gad2-IRES-Cre;R26R-EYFP* mice was recorded in their home cage by infrared video with concurrent EEG/EMG recordings for 2 h after dosing. Using the positions extracted from the video, we calculated the instantaneous speed of locomotion and then an average speed per 4-s epoch for each recording. When W epochs were compared, the overall range of values for mean locomotor velocity per epoch (0-16 cm/s) was similar for both treatments. However, the probability distribution of velocities from all mice under the two treatments were significantly different (*p* < 10^−308^, *n =* 14,067 and 11,016 epochs of W for CNO and SAL treatments, respectively, two-sample Kolmogorov–Smirnov goodness-of-fit hypothesis test). CNO-injected mice showed a trend toward spending more time at velocities above 2 cm/s, while SAL-injected mice spent more time at speeds below 2 cm/s (Fig. 3*F*). The mean W speed after SAL injection was 0.9 ± 0.2 and 1.7 ± 0.3 cm/s after CNO (*p* = 0.046, *n* = 9, *t* test). Thus, LH activation tends to increase activity by changing the proportion of slow versus fast speeds rather than by causing the mice to move at an abnormal speed in its home cage.

From hippocampal electrode recordings, it is well established that rodents increase θ power during exploration and active behavior (Vanderwolf, 1969; Shin et al., 2001; Hinman et al., 2011; Bender et al., 2015). We observed a strong correlation between HθWP and movement velocity when measuring cortical EEG. Figure 3*G* shows the temporal dynamics of mean locomotor speed per epoch (black trace) and scale-adjusted HθWP (green trace) for a mouse injected with CNO. The mean Pearson correlation between speed and HθWP after SAL and CNO injection were 0.44 ± 0.04 and 0.36 ± 0.07, respectively, reflecting a highly significant correlation in both cases (mean *p* < 10^−39^). No seizure-like activity or any sign of abnormal behavior was observed in CNO-injected animals, except for an increase in active wakefulness that was characterized by the same physiologic parameters of locomotor speed and EEG power that occurred during active periods after SAL injection.

### CNO-induced wakefulness is independent of Hcrt signaling

The nonspecific LH neuron transfection that resulted in the potent W-promoting effects after CNO injection at ZT12 (Fig. 2*A*,*A’* ) might be due to activation of Hcrt neurons. As a first step to evaluate this possibility, we administered the dual orexin receptor antagonist ALM to *Gad2-IRES-Cre;R26R-EYFP* mice at a high dose (200 mg/kg, i.p.) and at a time of day (ZT12) at which we had previously shown ALM to be sleep-inducing in other rodent strains (Morairty et al., 2012; Black et al., 2013). As in our previous studies, ALM increased the time spent in NREM sleep and decreased both the W time percentage and W bout duration, in this case, for >5 h relative to VEH (data not shown). Thus, ALM effects on sleep architecture were the inverse of those observed in response to LH activation and had a similar time course.

**Figure 4.**
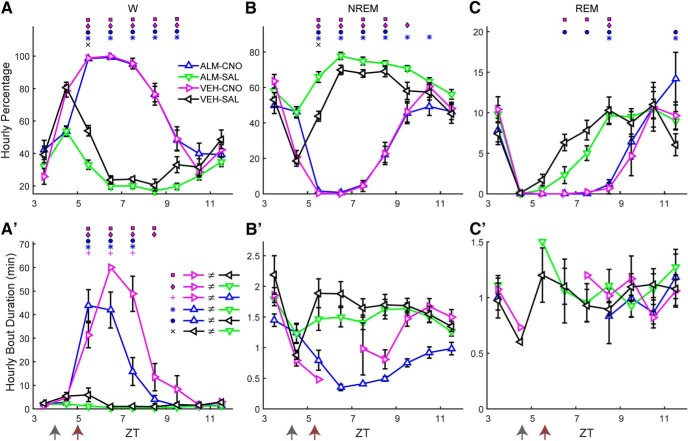
Hcrt signaling is not required for LH activation-induced arousal. Upper panels show hourly percentage of time spent in W (***A***), NREM (***B***), and REM sleep (***C***) and lower panels (***A’–C’***) show corresponding mean hourly bout duration for each state after injection of either VEH or ALM during the inactive phase at ZT4 followed by either SAL or CNO at ZT5. Arrows at the bottom left of the abscissa in ***A’–C’*** indicate the times of dosing. Symbols above each time point denote significant hourly differences between the indicated treatments according to the legends in panels ***A***, ***A’*** when a significant treatment × time interaction was found (*p* < 0.001, *n =* 10, two-way repeated measures ANOVA, Bonferroni *post hoc t* test).

To further test whether Hcrt signaling was required for the observed increase in W time after LH activation and whether time-of-day affects CNO-induced W promotion, we performed a dual-dosing study in which mice were treated with either VEH or ALM (200 mg/kg, i.p.) during the inactive period at ZT4 and, 1 h later, injected with either CNO (3 mg/kg, i.p.) or SAL while EEG and EMG were continuously recorded from ZT3 to ZT12. Figure 4 shows the percentage of time spent in each behavioral state (upper panels) and the mean bout duration of each state (lower panels), revealing a strong wake-promoting effect of CNO injection even in the presence of Hcrt receptor blockade by ALM (blue line). When all time points after dosing (ZT5-ZT12) were analyzed, significant treatment × time interactions were identified for the percentages of W (*F*_(18,162)_ = 31.1, *p* < 0.001; Fig. 4*A*), NREM (*F*_(18,162)_ = 32.4, *p* < 0.001; Fig. 4*B*), and REM sleep (*F*_(18,162)_ = 5.4, *p* < 0.001; Fig 4*C*; *n =* 10, two-way repeated measures ANOVA). There were no differences between the ALM-CNO and VEH-CNO treatments in the percentage of any state. However, the mean W bout duration, for which there was a significant treatment × time interaction (*F*_(18,162)_ = 17.4, *p* < 0.001), was significantly shorter in the ALM-CNO compared to the VEH-CNO treatment. These results indicate that, despite the strong wake-promoting effect of LH neuron activation produced by CNO injection, ALM reduced the mean W bout duration (Fig. 4*A’* ) due to occasional NREM episodes that interrupted sustained W bouts but were too brief (<12 s) to constitute a NREM bout. Due to the absence of sleep bouts from most mice after CNO injection, no statistical analysis was performed on sleep bout durations (Fig. 4*B’*,*C’* ).

**Figure 5.**
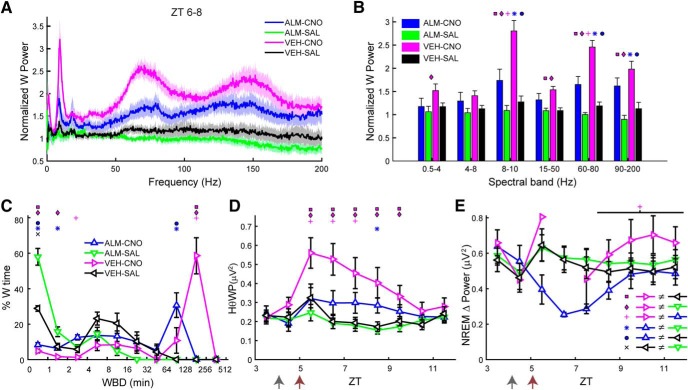
LH activation at ZT5 increases spectral power in the Hθ and γ bands of the EEG during W. ***A***, Normalized EEG power spectrum during W averaged from ZT6 to ZT8 for the different experimental treatments. ***B***, Mean power in the spectral bands of the EEG after treatment, normalized to baseline. ***C***, Time-weighted frequency histograms showing the proportion of wake bouts of each duration (WBD) relative to the total amount of wakefulness from ZT5 to ZT10 for the different experimental treatments. ***D***, HθWP for the different experimental treatments. ***E***, NREM δ power for all treatments. The discontinuity in the VEH-CNO line between ZT5 and ZT8 is because there were no sleep bouts during ZT6–ZT7. Horizontal bar denotes a significant effect of drug treatment and symbol above it indicates that the ALM-CNO versus VEH-CNO comparison was significantly different over this period (*p* = 0.024, *n =* 10, two-way repeated measures ANOVA, Bonferroni *post hoc t* test). Arrows at the bottom left of the abscissa in ***D***, ***E*** indicate the times of dosing for VEH or ALM at ZT4 and for SAL or CNO at ZT5. Symbols in ***B–D*** denote significant differences between the indicated treatments (*p* < 0.05) according to the legends in ***C***, ***E*** when ANOVA revealed significant interaction between drug treatment and power band.

In contrast to the active period (Fig. 3*D*), LH activation during the inactive period induced a large increase in the EEG power spectrum, especially in the Hθ and >50-Hz ranges (Fig. 5*A*). When the normalized power values during W were analyzed by conventional spectral bands, a significant treatment × power band interaction was found (*F*_(15,135)_ = 16.3, *p* < 0.001, *n =* 10, two-way repeated measures ANOVA), as depicted in Figure 5*B*, where the symbols above the bars denote significant differences between pairs of conditions according to the legend in Figure 5*E*. The normalized power for the VEH-CNO treatment (magenta) was significantly greater than that observed after VEH-SAL treatment (black) for the Hθ, Lγ, Hγ, and Vhγ bands by 120%, 42%, 107%, and 76%, respectively.

The distribution of W bout durations (Fig. 5*C*) differed for most treatments during the first 5 h after dosing (ZT5–ZT10). Pairwise comparison of the W bout duration distribution between treatments using the two-sample Kolmogorov–Smirnov goodness-of-fit hypothesis test with Bonferroni correction showed that all but the VEH-SAL versus ALM-SAL comparison (*p* = 0.51) were significantly different (*p* < 0.0073, number of bouts = 636, 755, 294, and 592 for ALM-CNO, ALM-SAL, VEH-CNO, and VEH-SAL, respectively). Figure 5*C* shows that dosing with ALM-SAL (green), not surprisingly, suppressed long W bouts; most of the time awake was spent in short W bouts. Conversely, most of the W time in the VEH-CNO treatment (magenta) was spent in long W bouts and the W bout distribution was shifted to the right. The W bout distribution for the ALM-CNO (blue) and VEH-SAL (black) treatments were intermediate between the other two treatments.

A strong treatment × time interaction was observed for the HθWP after dosing (*F*_(12,108)_ = 5.3, *p* < 0.001, *n =* 10, two-way repeated measures ANOVA). *Post hoc* analysis revealed that the VEH-CNO treatment was significantly different from the other three treatments during several hours, as depicted by the symbols above the respective ZT points in Figure 5*D*. VEH-CNO dosing (magenta) enhanced HθWP but this effect was suppressed when combined with ALM (ALM-CNO; blue), which differed from the ALM-SAL treatment (green) at one time point only; there was no difference between treatments in the absence of CNO.

To determine the homeostatic response to CNO-induced W during the inactive period, we compared NRD from the first time point after dosing when the average NREM time was at least 20% within an hour (which occurred after ZT8). A main effect of drug treatment on NRD was found (*F*_(3,24)_ = 3.7, *p* = 0.024, *n =* 9, two-way repeated measures ANOVA) without a treatment × time interaction (*F*_(9,72)_ = 0.74, n.s.). Figure 5*E* shows that NRD after VEH-CNO (magenta) was higher than after ALM-CNO (blue), despite similar amounts of prior W after both treatments (Fig. 4*A*).

### CNO-induced arousal is likely due to activation of a novel LH wake-promoting neuronal population

The currently known wake-promoting populations in the LH are the Hcrt neurons and two groups of GABAergic neurons (Herrera et al., 2016; Venner et al., 2016). To determine whether these wake-promoting cells were involved in the strong wake promotion described above, at least one week after the last dosing experiment, *Gad2-IRES-Cre;R26R-EYFP* mice received either CNO or SAL at ZT3 followed by 90 min of sleep deprivation (SD) by cage tapping or movements of the nesting material in their home cages and were then perfused transcardially with 4% PFA. SD was necessary because CNO-treated mice spent all 90 min awake while mice injected with SAL at ZT3 sleep for a large proportion of the 90 min after injection, which would result in differences in c-FOS expression that would be due to the difference in behavioral state rather than to activation by CNO. Since mice spend most of the time asleep between ZT0 and ZT3 and thereby discharge homeostatic sleep drive, SD can be performed for 90 min after ZT3 with minimal intervention. Consequently, SD for 90 min beginning at ZT3 allowed us to compare c-FOS expression during wakefulness with or without hM3Dq activation of LH neurons.

**Figure 6.**
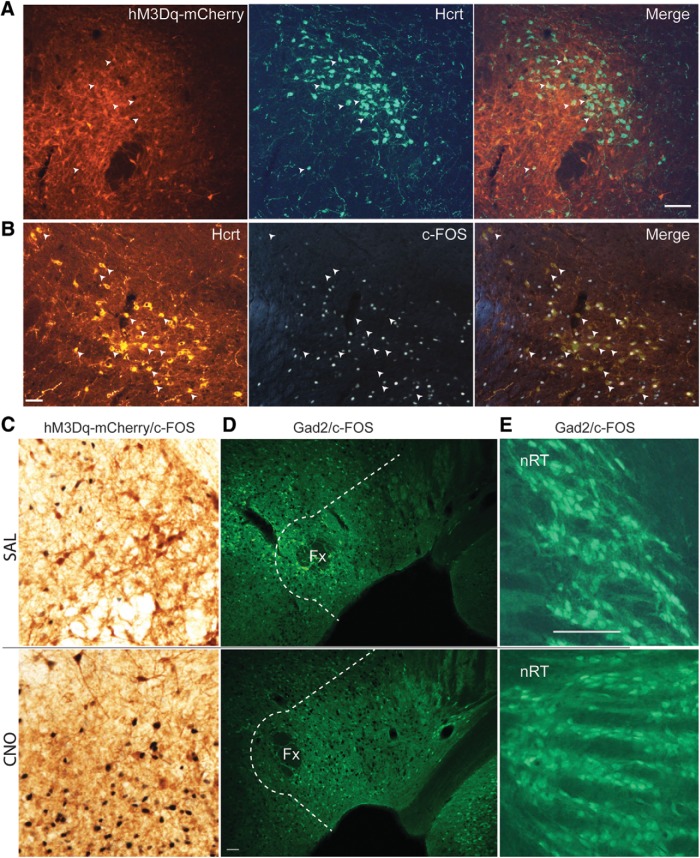
Sparse activation of Hcrt and inhibitory neurons after CNO injection in *Gad2-IRES-Cre;R26R-EYFP* mice. ***A***, Microphotographs showing hM3Dq-mCherry labeling in red (left), Hcrt neurons in green (center), and the merged images (right) in *Gad2-IRES-Cre;R26R-EYFP* mice transfected with AAV-TetO-hM3Dq-mCherry. White arrowheads denote Hcrt neurons coexpressing hM3Dq-mCherry. Scale bar = 100 µm. ***B***, Microphotographs showing Hcrt immunostaining (left), c-FOS staining (center), and the merged images (right) in AAV-injected *Gad2-IRES-Cre;R26R-EYFP* mice. White arrowheads denote Hcrt neurons that do not express c-FOS. Scale bar = 50 µm. ***C***, Photomicrographs of DAB immunohistochemistry of transfected neurons in LH (brown) and c-FOS expression (black nuclei) after SAL (upper panel) or CNO (lower panel) injection. ***D***, Photomicrographs of GFP-labeled LH inhibitory neurons (green cells) and c-FOS expression (black nuclei) in LH after SAL injection (upper panel) or CNO injection (lower panel). All hypothalamic cells lateral to the dotted white line were included in the Gad2/c-FOS counts. Scale bar = 50 μm. ***E***, GFP-labeled inhibitory neurons in the nRT (green cells) and c-FOS expression (sparse black nuclei) after SAL injection (upper panel) or CNO injection (lower panel). Scale bar = 100 µm (same scale as ***C***).

Brain tissue was sectioned in 30-μm coronal slices, divided into six series and processed for expression of the immediate early gene c-FOS as an indicator of neuronal activity and for expression of Hcrt, EYFP and mCherry. To determine whether hM3Dq was expressed in Hcrt neurons, we performed double immunohistochemistry on 4 *Gad2-IRES-Cre;R26R-EYFP* mice by coincubation with anti-Hcrt1/orexin-A and anti-Hcrt2/orexin-B antisera followed by an mCherry (rabbit anti-RFP) antibody which reported hM3Dq expression. Of the 627 ± 21 Hcrt cells found on average in each mouse, 14 ± 2% also expressed mCherry as depicted in Figure 6*A*, in which only six Hcrt neurons are also labeled by mCherry (white arrows).

Together with the results shown in Figure 1, the data above can be used to estimate the approximate percentage of transfected cells that are Hcrt neurons. Because we counted on average 1161 hM3Dq-transfected neurons in each tissue series, we can expect to find ∼7000 transfected cells in each *Gad2-IRES-Cre;R26R-*EYFP mouse. Because AAV expression occurred in ∼14% of Hcrt cells and, assuming there are ∼ 4000 Hcrt neurons in a mouse (McGregor et al., 2017), we estimate that ∼560 Hcrt neurons were transfected in each mouse which corresponds to <8% of the total number of transfected neurons.

Although Hcrt cells were not directly activated by hM3Dq and ALM dosing did not decrease the wake-promoting effects of CNO, it is still possible that Hcrt cells were indirectly activated and promoted arousal via glutamatergic excitation of its targets. To determine whether CNO causes a robust increase in c-FOS expression in Hcrt cells, we counted the number of Hcrt neurons expressing c-FOS after either CNO or SAL injection to assess whether they were indirectly activated as a downstream target. We counted on average 353 ± 70 Hcrt neurons per mouse in the CNO group (1766 Hcrt neurons in total), in which 68.8 ± 5.5% were FOS^+^, compared to 429 ± 38 Hcrt neurons/mouse in the SAL group (2143 Hcrt neurons in total), in which 54.8 ± 5.7% were FOS^+^ (*p =* 0.119, *n =* 5, *t* test). Thus, CNO injection did not cause a significant increase in c-FOS expression in Hcrt neurons during W in *Gad2-IRES-Cre;R26R-EYFP* mice. Figure 6*B* shows the Hcrt field and the negative image of c-FOS staining using DAB for a mouse injected with CNO 90 min before perfusion. Arrows indicate Hcrt neurons that did not express c-FOS and show that, despite the strong wake-promoting effects of CNO, a large percentage of Hcrt neurons was not activated.

Although the majority of transfected neurons by the AAV-TetO-hM3Dq-mCherry construct were not inhibitory, it is possible that CNO injection may activate either directly, or indirectly via downstream activation, one or both of the wake-promoting inhibitory neuronal populations recently described in the LH. In this case, the CNO-activated LH cells might project to and inhibit thalamic reticular nucleus (nRT) neurons (Herrera et al., 2016) and/or wake-promoting neurons located around and lateral to the fornix that are thought to project to and inhibit the ventrolateral preoptic area (VLPO; Venner et al., 2016). We reasoned that, if the mechanism of arousal observed in this study was due to activation of either of these pathways, there should be evidence of either strong activation of inhibitory neurons around the fornix or a suppression of activity in the nRT after activation of hM3Dq-transfected neurons. Figure 6*C* illustrates that CNO induced robust activation of hM3Dq-expressing neurons in the perifornical area, with c-FOS in black and mCherry (the hM3Dq reporter) in brown after SAL (upper panel) versus CNO (lower panel) injection.

To determine whether the pathways mentioned above were recruited after CNO injection, we performed YFP/c-FOS double immunohistochemistry in the same cohort of mice studied above. The number of double-labeled neurons in both hemispheres were counted from two slices from each of 10 *Gad2-IRES-Cre;R26R-EYFP* mice, sectioned around -1.7 mm from bregma. A ∼100-μm perimeter plus an area at an ∼45° angle from this perimeter laterally to the edge of the hypothalamus in the dorsal and ventral directions was delineated for cell counts. Figure 6*D* illustrates that this delineated area (white dotted line) includes both the perifornical area and the LH. In this panel, inhibitory neurons are in green and c-FOS expression in black from mice injected either with SAL (upper panel) or CNO (lower panel) showing that few Gad2^+^ neurons exhibited c-FOS expression. The average number of double-labeled inhibitory cells in this hypothalamic area did not differ between groups (48.4 ± 13 c-FOS^+^ cells per mouse after SAL injection, 242 double-labeled cells in total vs 75 ± 24.4 c-FOS^+^ cells after CNO injection, 375 cells in total; *p* = 0.36, *t* test, *n =* 5). In both conditions, the majority of inhibitory neurons did not express c-FOS; consequently, we did not calculate the percentage of double-labeled cells among all inhibitory neurons.

Similarly, Figure 6*E* shows the expression of c-FOS in the nRT after systemic injection of either SAL (upper panel) or CNO (lower panel). c-FOS-labeled inhibitory cells were identified bilaterally in coronal slices of the nRT region at 1.4–1.8 mm from bregma in one slide per mouse. In both treatments, c-FOS expression was very rare; thus, the percentages were not calculated and only the total number of double-labeled cells was counted (53.4 ± 9.8 cells per mouse after SAL injection, 267 double-labeled cells in total vs 55 ± 8 cells per mouse after CNO injection, 275 double-labeled cells in total; *p* = 0.9, *t* test, *n =* 5).

**Figure 7.**
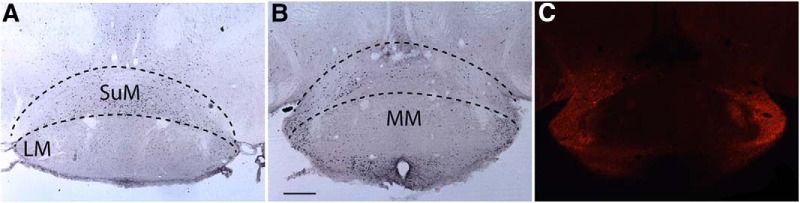
c-FOS activity in the SuM does not increase after CNO treatment. ***A***, c-FOS expression in the mammillary region after treatment with SAL followed by 90 min of SD. ***B***, c-FOS expression in the SuM after CNO treatment showing similar c-FOS expression than SAL treatment. ***C***, mCherry immunohistochemistry for the region depicted in ***B*** showing hM3Dq expression only in the lateral regions and almost no labeling in the medial aspect of SuM. Black dotted lines indicate the approximate region used for counting c-FOS in the SuM. LM, lateral mammillary nucleus; MM, medial mammillary nucleus. Scale bar = 250 μm.

The volume of AAV injected (340 nl) was intended to ensure transfection of the entire LH; the injections spread ventrally and dorsally up to the mammillary nucleus. Recently, it was found that chemogenetic activation of glutamatergic neurons in the supramammillary nucleus (SuM) promote wake without increasing locomotion, yet all EEG parameters indicative of active wake are increased (Pedersen et al., 2017). To determine whether CNO treatment caused a significant increase in c-FOS expression in SuM neurons, we also counted c-FOS expression in this region in 4 mice treated with SAL (a total of 2732 cells expressing c-FOS, 683 ± 64 cells/mouse) and 5 mice treated with CNO (3421 cells in total, 684 ± 61 cells/mouse) but found no evidence of a significant difference in c-FOS expression in the SuM between treatments (*p* = 0.99, *t* test, *n =* 4 and 5). Figure 7 illustrates these results, showing similar or even greater c-FOS expression in a mouse treated with SAL (Fig. 7*A*) compared to a mouse treated with CNO (Fig. 7*B*). Although some hM3Dq transfection was observed in this region, it was mostly on the edge of the lateral mammillary nucleus and not in the SuM itself (Fig. 7*C*).

### Dissection of Hcrt versus non-Hcrt arousal pathways

Figure 4*A* demonstrated that LH activation can evoke potent arousal during the inactive phase even in the presence of Hcrt receptor blockade, and Figure 6*A*,*B* shows that Hcrt neurons are neither directly nor indirectly involved in the potent wake promotion observed. However, this unknown LH wake-promoting population might have a downstream mechanism for wake promotion similar to that of the Hcrt neurons, i.e., these neurons may project to and excite known wake-promoting targets such as the TMN and LC. On the other hand, the observed wake-promoting action might be exerted via a different, parallel wake-promoting pathway yet to be described. We hypothesized that, if the wake-promoting pathway activated here overlaps with the Hcrt pathway, coactivation of both neuronal populations should trigger only minor differences when compared to activation of non-Hcrt neurons alone. On the other hand, if it is possible to further increase W after coactivation of Hcrt neurons, that would imply that Hcrt and non-Hcrt pathways are independent of each other.

**Figure 8.**
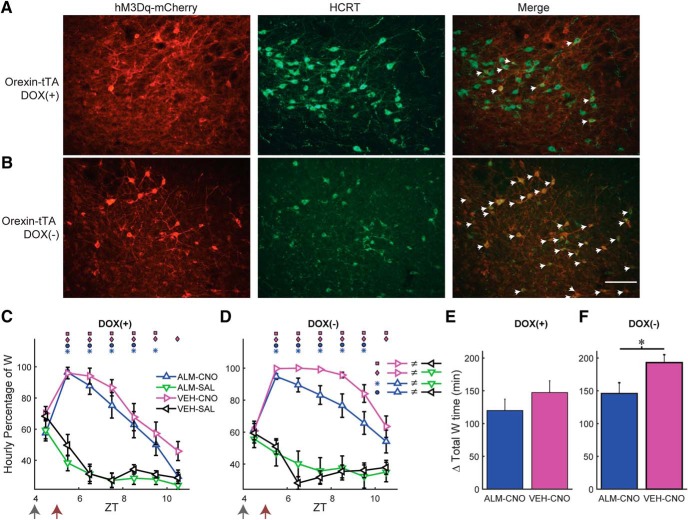
Dietary DOX regulates Hcrt neuron transfection and duration of W promotion in *Orexin-tTA* mice. ***A***, ***B***, Photomicrographs showing hM3Dq-mCherry labeling of transfected cells in red (left), Hcrt neurons in green (center), and the merged images (right) for *Orexin-tTA* mice in the DOX(+) (***A***) and DOX(-) (***B***) conditions. White arrows denote Hcrt neurons coexpressing hM3Dq-mCherry. Scale bar = 100 µm. ***C***, ***D***, Hourly percentage of time spent in W after injection of either VEH or ALM at ZT4 and either SAL or CNO at ZT5 in the DOX(+) (***C***, *n =* 11) or DOX(-) (***D***, *n =* 13) conditions. Symbols above each time point denote significant hourly differences (*p* < 0.05) between the indicated pairs of treatments according to the legends in ***A***, ***B*** when a significant interaction between drug treatment and time was found (*p* < 0.001, two-way repeated measures ANOVA, Bonferroni *post hoc t* test). Arrows at the bottom left of the abscissa indicate the times of dosing for VEH or ALM at ZT4 and for SAL or CNO at ZT5. ***E***, ***F***, Increase in total W time after dosing for ALM-CNO and VEH-CNO treatments relative to the VEH-SAL treatment in the DOX(+) (***E***, *p* = 0.29, *t test*, *n =* 11) and DOX(-) (***F***, *p* = 0.029, *t* test, *n =* 13) conditions.

To distinguish between these possibilities, we took advantage of the tTA dependence of the AAV-TetO-hM3Dq-mCherry construct. When Hcrt neurons that express tTA are exposed to this AAV, a large percentage would be expected to become transfected in addition to the ectopic expression depicted in Figure 1. Therefore, by adding or removing DOX from the mouse diet, the expression of hM3Dq in the tTA population can be manipulated. Consequently, we injected AAV-TetO-hM3Dq-mCherry bilaterally into the LH of *Orexin-tTA* mice that expressed tTA only in the Hcrt neurons. When *Orexin-tTA* mice were fed with DOX chow, ectopic expression occurred to a similar degree to that observed in *Gad2-IRES-Cre;R26R-EYFP* mice (Fig. 1*B*), but expression of hM3Dq-mCherry within Hcrt neurons was suppressed, as depicted in Figure 8*A*, where little overlap exists between the Hcrt neurons and hM3Dq-mCherry-expressing cells (denoted by eight white arrows). When DOX was removed from the chow for at least three weeks, in addition to the ectopic expression, transfected Hcrt neurons also expressed hM3Dq and could therefore be activated by CNO. Figure 8*B* shows that, in the absence of DOX, the hM3Dq-mCherry transgene is strongly expressed in the Hcrt neurons, denoted by 32 white arrows. In a series of coronal sections separated by 120 µm in three mice that had been fed with DOX chow for at least four weeks, there were 447 ± 17 Hcrt neurons per mouse (1340 neurons in total) of which only 18.0 ± 5.0% of Hcrt neurons expressed hM3Dq-mCherry. In contrast, in three mice that were fed with normal chow, there were 315 ± 112 Hcrt neurons per mouse (946 neurons in total), of which 62.2 ± 13.2% of Hcrt neurons expressed hM3Dq-mCherry. Thus, the absence of DOX in the diet enhanced expression of hM3Dq-mCherry in Hcrt neurons by >3-fold.

To study the effect of LH activation with or without concurrent Hcrt activation, we performed the same dosing study as in Figure 4, but we used *Orexin-tTA* mice fed with either normal chow or DOX chow. Although we injected AAV-TetO-hM3Dq-mCherry in 21 *Orexin-tTA* mice, seven mice did not show high levels of ectopic expression nor a significant increase in W during the first 2 h after CNO injection and therefore were excluded from analysis. From the 14 remaining mice, one was studied only under the DOX(+) condition, three were studied only in absence of DOX, and the remaining 10 mice were studied under both the DOX(+) and DOX(-) conditions. In these 10 mice, the second dosing study occurred at least three weeks after removal of DOX from the chow. As in the study illustrated in Figure 4, we injected either ALM or VEH at ZT4 and either CNO or SAL at ZT5 while continuously measuring EEG/EMG from ZT4 to ZT11.

In the DOX(+) condition when only a small percentage of Hcrt cells expressed hM3Dq, both VEH-CNO and ALM-CNO treatments evoked a strong increase in W time in a similar manner to that observed in *Gad2-IRES-Cre;R26R-EYFP* mice (Fig. 4*A*) and a strong treatment × time interaction occurred (*F*_(15,150)_ = 8.5, *p* < 0.001, *n =* 11, two-way repeated measures ANOVA) as depicted in Figure 8*C*. VEH-CNO (magenta) and ALM-CNO (blue) treatments did not differ from each other but they were significantly different from VEH-SAL (black) and ALM-SAL (green) for most of the time points. In the DOX(-) condition in which ∼62% of Hcrt cells were transfected, a similar result was observed, i.e., strong wake-promoting effects after CNO dosing regardless of the presence of ALM and a significant treatment × time interaction (*F*_(15,180)_ = 5.6, *p* < 0.001, two-way repeated measures ANOVA). A trend toward a stronger and longer-lasting W promotion was evident for the VEH-CNO (magenta) compared to ALM-CNO treatment (blue; Fig. 8*D*) but, since there was also a large amount of W in presence of ALM, the difference was not significant, likely due to a ceiling effect.

To further analyze the effect of dietary DOX and the difference in W promotion between the ALM-CNO and VEH-CNO treatments, we calculated the cumulative increase in W time for VEH-CNO and ALM-CNO treatments relative to the VEH-SAL treatment in both the DOX(+) (Fig. 8*E*) and DOX(-) (Fig. 8*F*) experimental groups after dosing. Whereas there was no significant difference between these treatments in the DOX(+) condition (*p* = 0.29, *n =* 11, *t* test; Fig. 8*E*), the concurrent activation of Hcrt neurons and other LH cells in the DOX(-) condition resulted in a significant difference in the W time increase in the VEH-CNO treatment relative to the ALM-CNO treatment (*p* = 0.029, *n =* 13, *t* test; Fig. 8*F*). CNO activation of Hcrt neurons might cause glutamate release by Hcrt neurons which could contribute to the W increase after the ALM-CNO combination. Comparing the blue bars in Figure 8*E*,*F*, DOX(-) mice exhibited 26.2 ± 14.1 min more W time than DOX(+) mice after ALM-CNO treatment but this difference was not significant (n.s., *n_1_* = 11, *n_2_* = 13, two-sample *t* test), suggesting a minor role for glutamatergic excitation in the observed increase in W time. A two-way unbalanced ANOVA revealed a significant effect of drug treatment (*F*_(1,1)_ = 5.47, *n_1_* = 11, *n_2_* = 13, *p* = 0.024) and type of chow (*F*_(1,1)_ = 5.11, *n_1_* = 11, *n_2_* = 13, *p* = 0.029), but no interaction between drug and type of chow (*F*_(1,44)_ = 0.38, n.s., two-way unbalanced ANOVA; Table 2).

**Table 2.** Results of two-way ANOVA for total increase in W time between DOX(+), DOX(-), ALM-CNO, and VEH-CNO drug treatment (Fig. 8)

Source	Sum Sq.	D.F.	Mean Sq.	*F*	*p*
Chow	15379.3	1	15379.3	5.11	0.035
Drug	16457.3	1	16457.3	5.47	0.0319
Chow*Drug	1138.4	1	1138.4	0.38	0.54
Error	132484.1	44	3011		
Total	166308.6	47			

## Discussion

Although the Hcrt cells are a small subset of the entire LH population, they are the only excitatory wake-promoting neurons currently known in this brain region and are thought to stabilize wakefulness via excitation of wake-promoting centers like the TMN, LC, and BF. Here, we have shown that chemogenetic excitation of LH neurons evokes sustained arousal in an Hcrt-independent manner. The existing literature, which has documented lethargy after suppression of neuronal activity in this area, supports the concept that LH neurons are essential to sustain wakefulness. The current results demonstrate that LH neuron activation promotes arousal without producing aberrant behavior or EEG anomalies, indicating that non-Hcrt neurons in LH could be part of the endogenous sleep-wake regulatory system. The fact that concurrent excitation of Hcrt neurons produced even a greater level of arousal (Fig. 8*D,F*) suggests that this putative novel wake-promoting system has a parallel, Hcrt-independent mechanism of action that could promote wakefulness via different downstream targets.

### LH neuron activation produces physiologic wakefulness without excessive hyperactivity

LH neuron activation during the active phase caused mice to remain awake almost continuously for 4 h (Fig. 2*A*), 1h longer than during the inactive phase (Fig. 4*A*), suggesting a circadian influence on W promotion. The change in sleep architecture underlying this increased wakefulness involved a shift toward longer duration W bouts (Figs. 3*A*, 5*C* ) rather than an increased number of W bouts.

The EEG during these sustained W bouts resembled the W EEG during the active phase, although the spectral power in the Hθ, Hγ, and Vhγ bands increased by 20–30% (Fig. 3*D*,*E*). During the inactive phase, LH activation increased spectral power in the Hθ band by 120% (Fig. 5*A*,*B*), which was directly correlated with the speed of locomotion (Fig. 3*G*) and large increases in spectral power also occurred in the γ range. It was shown recently that Hcrt as well as GABAergic neurons in LH directly regulate locomotor behavior (Kosse et al., 2017; Perez-Leighton et al., 2017; Qualls-Creekmore et al., 2017) and that hippocampal neurons that give rise to the θ rhythm observed in the EEG during locomotion can activate inhibitory neurons in the lateral septum that, in turn, inhibit LH neurons and thereby reduce locomotor speed (Bender et al., 2015). Therefore, activation of LH neurons might activate circuits that regulate locomotor activity itself and not necessarily arousal. Strong activation of these circuits should evoke non-physiologic behaviors due to the potent activation observed after CNO treatment in hM3Dq-transfected cells (Fig. 6*C*). Nevertheless, despite the large increase in HθWP after VEH-CNO treatment (Fig. 5), the maximum absolute values (between 0.5 and 0.6 μV^2^) were similar to those observed during the active phase suggesting that mice were as active after CNO injection at ZT5 as during active wake after ZT12 and, therefore, that the changes in locomotor activity are due to changes in behavioral state rather than to manipulation of neuronal circuits that regulate locomotion. The range of velocities observed in mice injected with CNO was similar to those observed after SAL injection; however, CNO-injected mice spent more time moving at higher speeds (Fig. 3*F*). Together, these findings suggest that LH activation after transfection with AAV-TetO-hM3Dq produces physiologically normal wakefulness.

To be part of the physiologic regulation of sleep and wake, wake-promoting neurons should not cause aberrant EEG patterns or pathologic behavior, should be active either during or at the onset of wake, and suppression of their activity should cause a decrease in arousal. Here, we have shown that activation of an unknown LH population can indeed cause active wake without aberrant behavior nor EEG anomalies, indicating that these LH cells could be considered part of the physiologic regulation of arousal. Future work will determine whether they are necessary and sufficient to sustain wake.

### Is the observed arousal caused by a novel hypothalamic wake-promoting neuronal population?

HA neurons in the TMN have been shown to be wake-promoting, yet immunohistochemistry revealed that this cell group was not transfected by our AAV (Fig. 1*E*) despite the presence of some transfected cells in this region of the hypothalamus. Two wake-promoting GABAergic neuronal populations have recently been described in the LH. Inhibitory nRT-projecting LH neurons have been shown to induce wakefulness on acute stimulation during NREM but not during REM sleep (Herrera et al., 2016), yet we showed that the amount of c-FOS expression in nRT was very low and there was no difference in c-FOS expression whether mice received CNO or SAL while being kept awake (Fig. 6*E*). Although the absence of FOS expression does not necessarily mean that neurons were inactive, it is unlikely that a further reduction in nRT activity mediated by LH GABAergic input after CNO dosing would trigger the robust wake-promoting response shown here. Similarly, VLPO-projecting inhibitory neurons located in the perifornical area have been proposed to promote wake by inhibiting the sleep-active inhibitory neurons in VLPO (Venner et al., 2016). Because only a minority of inhibitory neurons in the perifornical area expressed c-FOS and the number of c-FOS^+^ cells did not change after CNO versus SAL injection (Fig. 6*D*), it is unlikely that the increase in W time and locomotor activity was caused by only sparse excitation of VLPO-projecting inhibitory neurons. Because ∼30% of transfected neurons in LH were GABAergic, it is possible that the CNO-activated LH population included some of these novel wake-promoting cell groups. Recently, glutamatergic wake-promoting neurons were found in the SuM region (Pedersen et al., 2017). Although we observed some transfected cells in this region, transfection was sparse and mostly lateral and ventral to the SuM (Fig. 7*C*). Since SuM neurons expressed significant c-FOS during wake regardless of treatment, the marginal increase on the edges, which seemed to cause an increase in c-FOS expression in the lateral mammillary nucleus, did not affect the overall expression of c-FOS in SuM. More importantly, mice were described in that study as often stationary after CNO treatment, there was no correlation between increase in Hθ activity and locomotion, and the EMG was similar to control levels during the inactive phase. These results are the opposite of what we obtained (Fig. 3*E*,*F*); thus, although some of these wake-promoting cells could have been activated, it is unlikely they are the main source of the observed arousal. Although more experiments will be required to demonstrate that LH wake-promoting neurons activated in the present study are a different population than currently known wake-promoting populations, our evidence points in that direction.

### Concurrent activation of LH wake-promoting neurons and Hcrt neurons enhances arousal

Because thousands of neurons were transfected in *Gad2-IRES-Cre;R26R-EYFP* mice which lack tTA (Fig. 1*C*,*D*), the AAV-TetO virus did not restrict expression to only tTA neurons. However, this partial tTA dependence enabled an interesting strategy to compare the physiologic effects of LH activation when different proportions of Hcrt neurons expressed the hM3Dq receptor. In mice maintained on a DOX(+) diet, 18% of Hcrt cells expressed hM3Dq-mCherry as well as a much larger number of non-Hcrt cells. There was no difference in the amount of wakefulness produced by CNO injection whether these mice received SAL or ALM pretreatment. In contrast, 62% of Hcrt neurons in DOX(-) mice expressed hM3Dq-mCherry and, in these mice, ALM pretreatment attenuated the increase in W time produced by CNO injection with respect to the VEH-SAL treatment. Thus, concurrent Hcrt and non-specific LH neuron activation produced more robust arousal as shown by the significant effect of Dox chow shown in Table 2. The lack of a significant interaction between chow and treatment can be attributed to ceiling effects.

Hcrt neurons are also glutamatergic although, to our knowledge, there is no direct *in vivo* evidence of a role for glutamate release by the Hcrt neurons in the regulation of sleep and wake. Because ALM blocks the excitation mediated by Hcrt peptides but not glutamatergic excitation, comparison of the increase in W time under the ALM-CNO treatment between the DOX(-) and DOX(+) conditions might reveal the magnitude of glutamatergic-mediated arousal. We suggest that glutamatergic excitation mediated by Hcrt neurons has a secondary role in wake promotion because the difference in W amount between these two conditions (Fig. 8*E* vs *F*, blue bars) was not significant.

### Potential therapeutic target for sleep disorders

During the inactive phase, the time-weighted frequency histograms for the ALM-SAL treatment shifted toward shorter W bout durations (Fig. 5*C*), reflecting an inability to sustain prolonged W bouts after Hcrt blockade that is reminiscent of the symptomatology of narcoleptic patients. In the ALM-CNO treatment, activation of the parallel LH arousal pathway induced prolonged W bouts despite blockade of Hcrt signaling which demonstrates that it is possible to achieve consolidated W in the absence of Hcrt signaling. Activation of this novel arousal pathway may also provide treatment for hypersomnia. Conversely, based on lesion and inactivation experiments performed by others, we hypothesize that suppression of the activity of this LH pathway promotes sleep, suggesting a potential treatment for insomnia. Future work will focus on the molecular neuroanatomical identification of these LH wake-promoting neurons, their activation and inhibition, as well as identification of their afferent inputs, efferent targets and the neurotransmitters involved.

## Synthesis

Reviewing Editor: Rae Silver, Columbia University Decisions are customarily a result of the Reviewing Editor and the peer reviewers coming together and discussing their recommendations until a consensus is reached. When revisions are invited, a fact-based synthesis statement explaining their decision and outlining what is needed to prepare a revision will be listed below. The following reviewer(s) agreed to reveal their identity: Clifford Saper, Giulio Tononi

This is an interesting series of experiments, suggesting, but not proving, that there is an additional population of wake-promoting neurons in LH distinct from the Hypocretin and GABAergic cells.

A major problem remains in that the authors have not acknowledged that there are a number of interpretations of the results. Among them that they have activated non-specifically at least two known lateral hypothalamic sources of wake-promoting cells.

-Error in citing the literature: The authors claim that they probably did not involve the lateral hypothalamic GABAergic neurons described by Venner et al. 2016, which cause wakefulness by inhibiting the ventrolateral preoptic nucleus, because they had relatively little cFos in the perifornical region. In fact, Venner et al. describe the neurons that they activated in the ventral 2/3 of the lateraly hypothalamus. Although a small percentage of these extend into the perifornical area, the vast bulk of the group is not in that location. So, examining cFos expression in the perifornical region is not an adequate way to eliminate their participation.

-The authors must quantify the involvement of hM3Dq and cFos expression in the supramamillary region and the ventrolateral LH in the Results section, and if there was significant involvement, they need to acknowledge that their results could be explained at least in part by the inclusion of these recently described cell groups in their injection sites. This is important as Pedersen et al. found strongly wake-promoting glutamatergic cells in the supramammillary region, it is important in this study to document the extent to which this area was included in the injection sites. the authors do not even mention this possibility until the discussion, where they claim (providing no data in the results section) that the transfected cells in the supramammillary region were mostly ventral and lateral, which they claim would not affect arousal. However, strongly arousing responses were obtained by Pedersen et al from across the supramammillary region, including its ventrolateral segment, and the only area that produced only mild arousing effects was the Vglut2/Vgat cells in medial part.

-The abstract should be edited to better reflect the limitation of the study. The abstract should clearly state that we know already of wake-promoting GABAergic cells in LH, and that this study is unable to completely rule out that this population account for the wake-promoting effects that were observed. As written, the abstract quotes the 18% of hypocretin cells, but not the 30% of CNO-activated cells that are GABAergic; there should be a sentence at the end stating that these cells could still account for the results.

-A visual abstract would be welcome
